# Selectivity of Sol-Gel and Hydrothermal TiO_2_ Nanoparticles towards Photocatalytic Degradation of Cationic and Anionic Dyes

**DOI:** 10.3390/molecules28196834

**Published:** 2023-09-27

**Authors:** Md. Torikul Islam, Md. Nahid Parvez Roni, Md. Yunus Ali, Md. Robiul Islam, Md. Shamim Hossan, M. Habibur Rahman, A. A. S. Mostofa Zahid, Md. Nur E Alam, Md. Abu Hanif, M. Shaheer Akhtar

**Affiliations:** 1 Department of Chemistry, University of Rajshahi, Rajshahi 6205, Bangladesh; 2 Bangladesh Atomic Energy Commission, Dhaka 1207, Bangladesh; 3 Institute of Carbon Technology, Jeonju University, Jeonju 55069, Republic of Korea; 4 Graduate School of Integrated Energy-AI, Jeonbuk National University, Jeonju 54896, Republic of Korea

**Keywords:** titanium dioxide, photocatalyst, sol-gel, hydrothermal, methylene blue, methyl orange, anatase, brookite phase

## Abstract

Titanium dioxide (TiO_2_) nanoparticles have been extensively studied for catalyzing the photo-degradation of organic pollutants, the photocatalyst being nonselective to the substrate. We, however, found that TiO_2_ nanoparticles prepared via the sol-gel and hydrothermal synthetic routes each possess a definite specificity to the charge of the substrate for photodegradation. The nanoparticles were characterized by SEM, FTIR, XRD, TGA, and UV-visible spectra, and the photocatalytic degradation under UV-B (285 nm) irradiation of two model compounds, anionic methyl Orange (MO) and cationic methylene blue (MB) was monitored by a UV-visible spectrophotometer. Untreated sol-gel TiO_2_ nanoparticles (T_sg_) preferentially degraded MO over MB (90% versus 40% in two hours), while after calcination at 400 °C for two hours (T_sgc_) they showed reversed specificity (50% MO versus 90% MB in one hour). The as-prepared hydrothermal TiO_2_ nanoparticles (T_ht_) behaved in the opposite sense of T_sg_ (41% MO versus 91% MB degraded in one and a half hours); calcination at 400 °C (T_htc_) did not reverse the trend but enhanced the efficiency of degradation. The study indicates that TiO_2_ nanoparticles can be made to degrade a specific class of organic pollutants from an effluent facilitating the recycling of a specific class of pollutants for cost-effective effluent management.

## 1. Introduction

Synthetic dyes are extensively used in dying textiles, leathers, papers, plastics, and fibers. However, most of the synthetic dyes are toxic, mutagenic, carcinogenic, and allergenic. Discharging the wastewater of these dyeing industries has instigated a major problem of water contamination as these dyes contain stable aromatic rings that resist degradation under normal conditions [1,2,3,4,5]. Thus, the removal of these organic contaminants from the effluents by degrading them into environmentally benign fragments is energy-intensive and costly. Accordingly, the cost-effective removal of synthetic dyes from the effluents to guard against their adverse effect on the environment is a big challenge. Selective degradation of some of the components from the effluents can pave the way for recycling and cost-effective management of the effluents of these industries.

Nanometric titanium dioxide (TiO_2_) is a chemically stable, strongly oxidizing, nontoxic, and environmentally benign photocatalyst material that has found applications in diversified fields, e.g., in the photodegradation of organic pollutants, hydrogen generation by water splitting, solar energy conversion, self-cleaning coating technology, in biosensors and bioimaging devices, and dye-sensitized solar cells to name a few [6,7,8,9,10,11,12,13]. The synthetic method is a crucial factor in the photocatalytic and structural properties of the nanoparticles. There are many synthetic routes to nano-TiO_2_, such as hydrothermal [14,15], sol-gel [16,17], solvothermal [18,19], direct oxidation [20,21], chemical vapor deposition [22] and microwave methods [23,24]. Each of these synthetic routes may produce TiO_2_ nanoparticles of different crystalline modifications including the amorphous phase.

TiO_2_ has three crystalline polymorphs such as anatase, rutile, and brookite, each of which may show different photocatalytic activity. Moreover, the chemical reactivity may strongly depend on the crystalline structure, morphology, size, and shape of the TiO_2_ nanoparticles of a particular crystalline polymorph [25,26,27,28]. It has been demonstrated that nanoscale TiO_2_ such as nano-spheres, nano-prisms, nano-wires, nano-rods, and nano-tubes having appreciably large specific surface areas lead to better physical and chemical properties [29], including catalyzing photo-degradation of organic pollutants. This makes it essential to compare and evaluate different synthetic methods for obtaining control over the size, shape, surface properties, and crystalline polymorph of the TiO_2_ nanoparticles for making the best use of their photocatalytic ability.

In this study, we have demonstrated that the TiO_2_ nanoparticles prepared by two different synthetic routes, such as sol-gel and hydrothermal are quite specific toward the photocatalytic degradation of cationic and anionic dyes under UV-B irradiation (285 nm). We have found that the photocatalytic degradation efficiency of untreated TiO_2_ nanoparticles prepared by the sol-gel technique is remarkably higher for the anionic model dye methyl orange (MO), compared to methylene blue (MB), a cationic dye (90% for MO versus 40% for MB degradation within two hours). The said specificity was reversed for the same nanoparticles calcined at 400 °C, especially in the early stage of the degradation reaction (50% for MO versus 90% for MB in one hour). On the other hand, the as-synthesized hydrothermal nanoparticles displayed specificity opposite of the as-synthesized sol-gel nanoparticles (91% for MB versus 41% for MO in one and a half hours).

## 2. Results and Discussion

### 2.1. Morphological, Structural, Photophysical, and Thermal Analysis of TiO_2_ Nanoparticles

**Figure 1:**
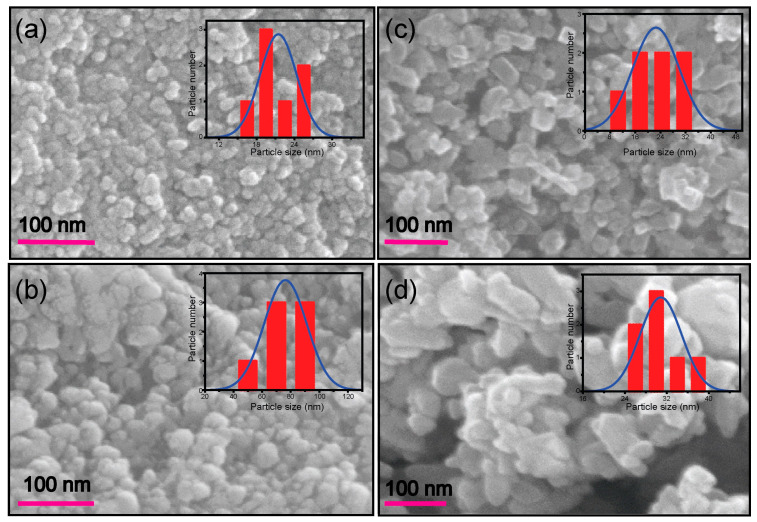
SEM images of T_sg_ (**a**), T_sgc_ (**b**), T_ht_ (**c**)_,_ and T_htc_ (**d**). Inserts represent the size-distribution curves (blue lines) of the respective nanoparticles.

The morphology of the TiO_2_ nanoparticles prepared by the sol-gel method: as-prepared (T_sg_), and calcined at 400 °C (T_sgc_) and by the hydrothermal method: as-prepared (T_sg_), and calcined at 400 °C (T_htc_) were observed by a scanning electron microscope (SEM) and the SEM micrographs are shown in Figure 1. From the SEM images, we observe that the nanoparticles synthesized by both methods were agglomerated and non-uniform in size. The size distribution curves of the T_sg_, T_sgc_, T_ht,_ and T_htc_ nanoparticles have been shown in the insets of Figure 1. The size range of the T_sg_, T_sgc_, T_ht,_ and T_htc_ nanoparticles were 17–26 nm, 68–97 nm, 12–32 nm, and 27–39 nm, respectively. The shape of T_sg_ and T_sgc_ were approximately spherical (Figure 1a,b) whereas that of T_ht_ and T_htc_ were approximately rectangular prism and cubic (Figure 1c,d), and the surfaces of all the nanoparticles were rough.

**Figure 2:**
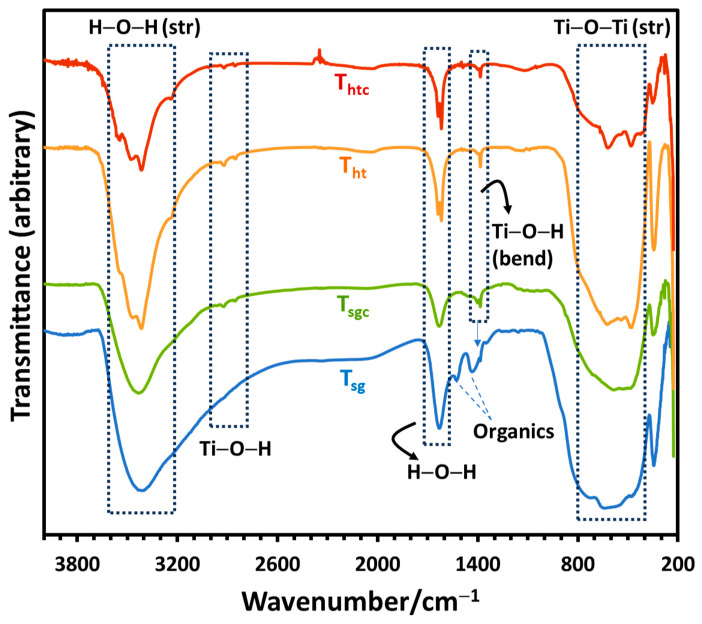
FTIR spectra of the synthesized photocatalysts as indicated in the legend.

Fourier transform infrared (FTIR) spectroscopy being a surface-sensitive analytical technique provides valuable information on the surface region of the nanoparticles. The FTIR spectra of the nanoparticles are displayed in Figure 2. These are typical of the TiO_2_ nanoparticles confirming their successful synthesis. The broad absorption band observed in the region 400–800 cm^−1^ originated from Ti–O and bridging Ti–O–Ti stretching vibrations, while the absorption peak at 1386 cm^−1^ is associated with the Ti–O bending vibrations. The absorption band at 3420 and 1615 cm^−1^ represented the –O–H stretching and bending vibrations, respectively, of water chemisorbed on the TiO_2_ nanoparticle surface. The two small absorption peaks at ~2915 and ~2842 cm^−1^ have been assigned to be associated with the Ti–OH bond vibrations [11,30]. The extra absorption peaks at ~1428 and ~1528 cm^−1^ found in as-prepared sol-gel TiO_2_ (T_sg_) can be attributed to the adsorbed organic materials on the surface of the nanoparticles during synthesis.

**Figure 3:**
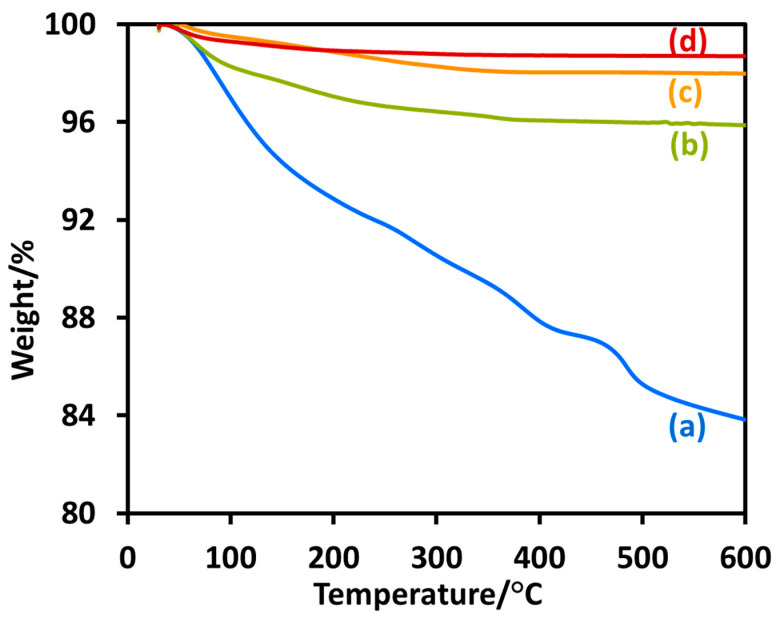
TGA traces of the synthesized nanoparticles T_sg_ (**a**), T_sgc_ (**b**), T_ht_ (**c**), and T_htc_ (**d**).

The adsorbed organic material on the surface of the nanoparticles was estimated by the thermogravimetric analysis (TGA) profiles of the nanoparticles (Figure 3). The TGA trace of T_sg_ exhibited three-stage weight loss typical for TiO_2_ nanoparticles [4,5,6,7,8]. The first ca. 6% weight loss below 200 °C mainly originated from the evaporation of physisorbed water and alcohols on the nanocomposite surface. The second weight loss of ca. 7% in the region 200 °C–500 °C for T_sg_ correlated mainly with the degradation of the organic components, which were adsorbed from the reaction mixture during synthesis. The third minor weight loss occurring above 500 °C was attributed to the loss of water produced by the condensation of neighboring terminal Ti–OH groups on the nanoparticle surfaces [10,11,12,13]. For the nanoparticles obtained after calcination of T_sg_ at 400 °C, the weight loss beyond 200 °C was minor, apparently due to the almost complete removal of the adsorbed organic materials during calcination. Only a slight weight loss (<1%) of hydrothermal T_ht_ and T_htc_ indicated that they were pure TiO_2_ with cleaner surfaces. The heavy loss of weight (ca. 16%) for the as-prepared sol-gel nanoparticles, T_sg_ indicated a high concentration of organic material adsorbed on the surface of this nanoparticle.

**Figure 4:**
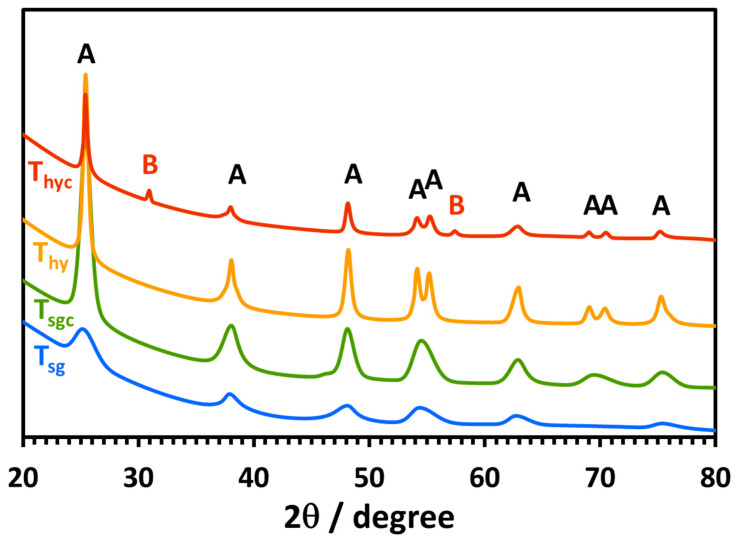
XRD patterns of the synthesized photocatalysts as indicated in the legend. Diffraction peaks corresponding to the anatase phase (A) and brookite phase (B) are also indicated.

The crystalline phases of the synthesized T_sg_, T_sgc_, T_ht,_ and T_htc_ nanoparticles were determined by X-ray diffraction (XRD), and the representative patterns are displayed in Figure 4. The precise Bragg diffraction peaks signify the crystalline nature of the nanoparticles. The Bragg diffraction peaks designated by (A) agreed with the anatase phase of TiO_2_ with tetragonal arrangement [31,32,33], especially, the two most intense diffraction peaks found in all of the samples, viz., at (2θ) 25.0° and 48.0° are typical of the anatase phase. This result indicates that all of the prepared TiO_2_ nanoparticles were predominantly of the anatase phase. As observed in SEM images (Figure 1), the sol-gel TiO_2_ nanoparticles were smaller in size, which resulted in relatively broad diffraction peaks and consequently alongside peaks were not resolved. The hydrothermal TiO_2_ nanoparticles T_ht_ prepared at 240 °C being larger, the diffraction peaks were well resolved and confirmed with the anatase polymorph. However, after calcination at 400 °C the hydrothermal TiO_2_, T_htc_, displayed two additional diffraction peaks (designated by B) at (2θ) ~31° and ~58°, the former of which is the (d_121_) peak of the brookite phase [34,35], confirming that T_htc_ is a mixture of anatase and brookite crystalline polymorphs of TiO_2_.

The degree of crystallinity (*λ*) of the synthesized TiO_2_ nanoparticles calculated from the XRD data [32] according to Equation (1),

(1) $$\lambda =\left[A_{c}/(A_{c}+A_{a})\right]\times 100\%$$

where $A_{c}$ is the area covered by the crystallization peaks and $\left(A_{c}+A_{a}\right)$ is the total crystalline and amorphous area calculated by OriginLab’s Origin software (version 2019b 64bit), is shown in Table 1. The crystal size (*l*) of the nanoparticles calculated from the XRD data according to the method of Scherrer [36] is also presented in Table 1. The XRD data revealed that the size of the sol-gel nanocrystals was much smaller than the hydrothermal nanocrystals. The size increased by about 40% and 24% after the calcination of the sol-gel and the hydrothermal nanocrystals, respectively. The degree of crystallinity of all the nanoparticles was low compared to the commercial Degussa (Evonik) P25 TiO_2_ photocatalyst (total crystallinity 92% in a typical sample) [37]. However, the degree of crystallinity was increased by 108% for the sol-gel nanoparticles while that of the hydrothermal nanoparticles decreased by 46% upon calcination. The decrease in crystallinity can be attributed to the fact that for the T_htc_ sample, which is a mix of anatase and brookite, the growth of the brookite phase at the expense of the anatase phase during calcination slowed down the crystallization process [38].

**Figure 5:**
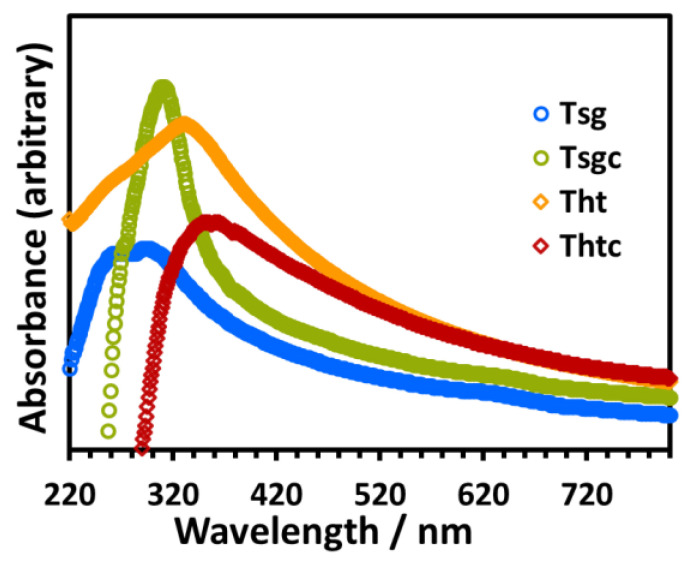
UV-visible spectra of stable sols (1 mg/10 mL) of the photocatalysts in ethanol, as indicated in the legends.

Figure 5 displays the UV-visible spectra of stable sols of the photocatalysts in ethanol. The $\lambda_{max}$ for T_sg_, T_sgc_, T_ht_, and T_htc_ were 300 nm, 313 nm, 335 nm, and 358 nm, respectively. Thus, there is a red shift of 13 nm and 23 nm of the $\lambda_{max}$ for the sol-gel and the hydrothermal nanoparticles, respectively, upon calcination. As the $\lambda_{max}$ is related to the absorption onset, it can be inferred that the optical bandgap of the materials significantly reduced upon calcination [39].

### 2.2. Photocatalytic Degradation Study

The catalytic efficiency of the nanoparticles for photodegradation of two model organic dyes, MB and MO was conducted at 10 °C under UV-B irradiation; a detailed procedure is given in Section 3.4. MB is cationic whereas MO is anionic. The choice of these model compounds was made to exploit their contrasting chemical nature for studying the selectivity of the nanoparticles for catalyzing the photodegradation of a particular class of compounds, and also because they have previously been extensively used as model compounds for photodegradation study [4,5,32].

#### 2.2.1. Control Experiment

**Figure 6:**
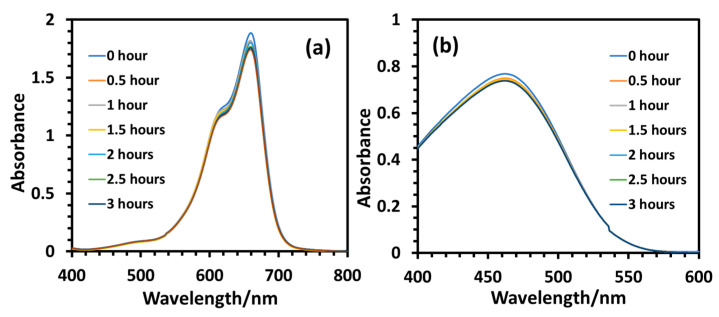
UV-visible absorbance data of the model dye solutions (10 mg/L) at various reaction times during photodegradation at 10 °C in a control experiment (without any photocatalyst) under UV-B irradiation: (**a**) MB, (**b**) MO.

A control experiment was conducted under identical experimental conditions without the presence of any photocatalyst, the data of which are presented in Figure 6. The intensity of absorption maximum of the dyes decreased only slightly during three hours of UV-B irradiation, displaying negligible degradation of the dyes without a photocatalyst (Figure 6a,b).

#### 2.2.2. Determination of the Optimum Dose of the Nanoparticles

**Figure 7:**
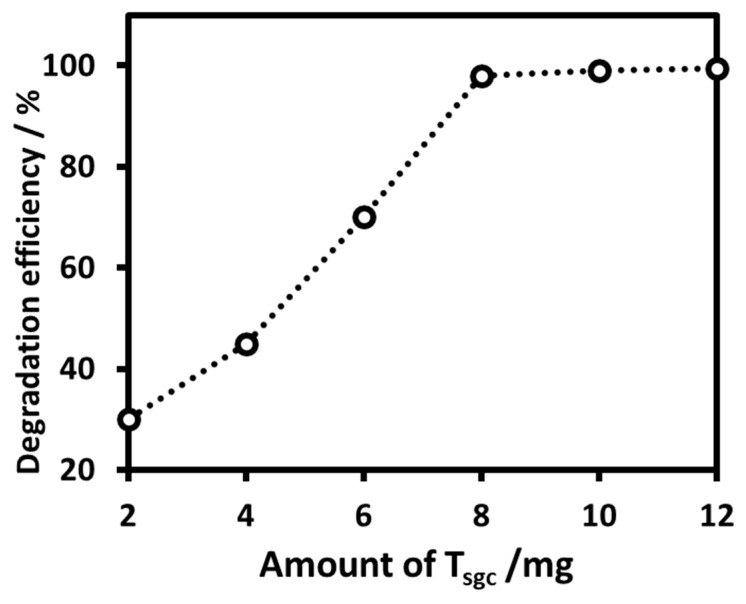
Effect of the dose of the photocatalyst (T_sgc_) on the degradation of MB under UV-B irradiation. 10 mL of a 10 ppm MB solution was irradiated for three hours in the presence of the photocatalyst.

Before starting a photocatalytic degradation experiment, we determined the minimum amount of the nanoparticle (adsorbent) that can degrade the maximum amount of the substrate. The experiment for dose effect was carried out in the presence of the nanoparticles under UV-B irradiation at 10 °C for three hours. Figure 7 displays the % degradation of the model dye MB as a function of the amount of the nanoparticles T_sgc_ as representative. The maximum degradation efficiency of MB (98%) was observed at the optimum dose of 8 mg/10 mL of the catalyst. Subsequent degradation experiments were conducted by using this optimum dose.

#### 2.2.3. Adsorption Study

**Figure 8:**
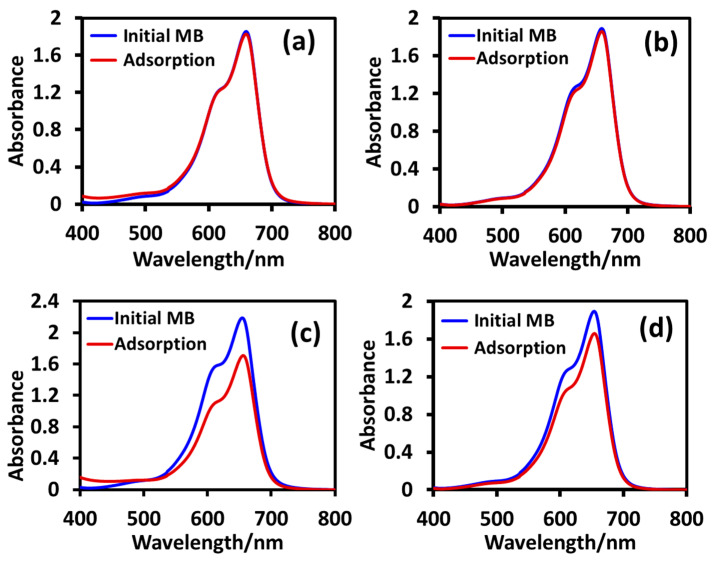
UV-visible absorption spectra of methylene blue before and after adsorption by T_sg_ (**a**), T_sgc_ (**b**), T_ht_ (**c**), and T_htc_ (**d**) at ambient temperature (25 °C).

**Figure 9:**
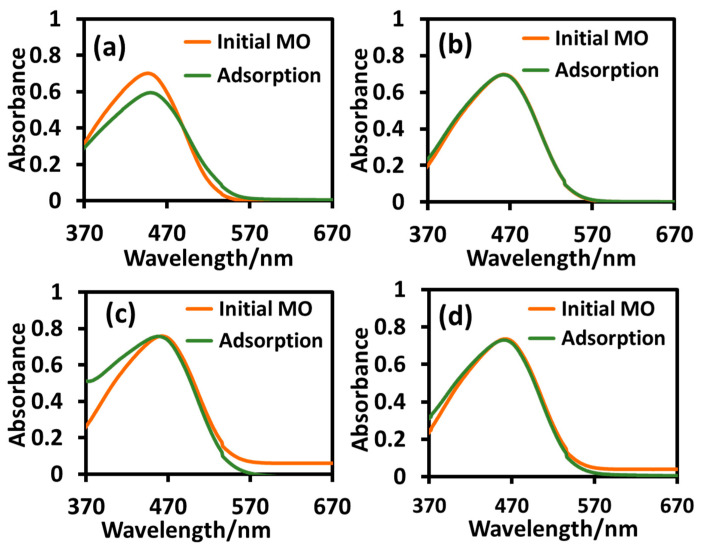
UV-visible absorption spectra of methyl orange before and after adsorption by T_sg_ (**a**), T_sgc_ (**b**), T_ht_ (**c**), and T_htc_ (**d**) at ambient temperature (25 °C).

We conducted an adsorption study using the optimum dose of 8 mg/10 mL of the nanoparticles T_sg_, T_sgc_, T_ht_, and T_htc_ on 10 ppm solutions of MB and MO under dark conditions at ambient temperature (25 °C) for 30 min and the data are accumulated in Figure 8 and Figure 9, respectively. As we can observe from Figure 8 that the adsorption capacity of the sol-gel T_sg_, and T_sgc_ nanoparticles for cationic MB was negligible, while that of the hydrothermal T_ht_ and T_htc_ was quite high at 26% and 13%, respectively. The corresponding data for the adsorption of anionic MO are shown in Figure 9 where it is clear that the adsorption capacity of all the nanoparticles studied was negligible for MO excepting T_sg_, which showed an appreciable adsorption of 16%.

#### 2.2.4. Photocatalytic Degradation of Cationic MB Dye under UV-B Irradiation

**Figure 10:**
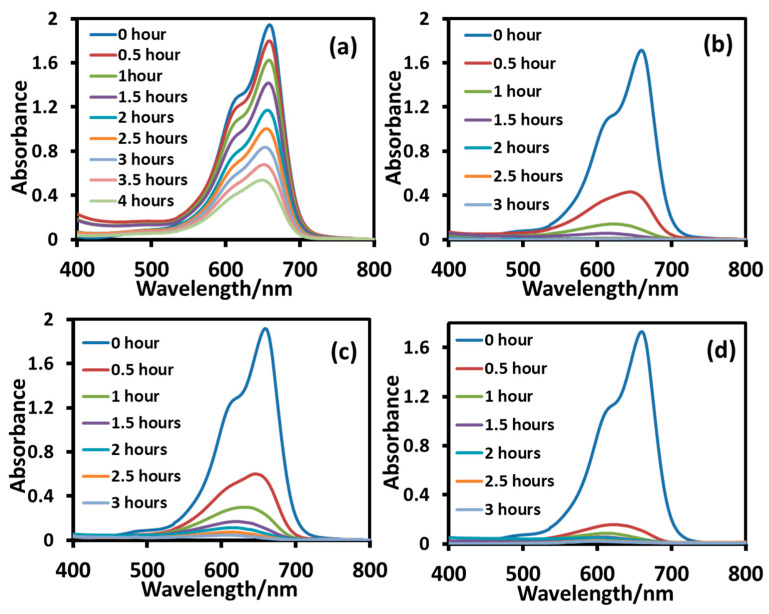
UV-visible absorption spectra of aqueous solutions of MB during degradation under UV-B irradiation in the presence of T_sg_ (**a**), T_sgc_ (**b**), T_ht_ (**c**), and T_htc_ (**d**). Reaction conditions: dye = 10 mg/L, nanoparticles = 8 mg/10 mL, temperature: 10 °C.

The photodegradation study of cationic MB was conducted in the presence of T_sg,_ T_sgc_, T_ht_, and T_htc_ under UV-B irradiation. The photocatalytic efficiency of the nanoparticles was determined by measuring the intensity of the optical absorption of MB in UV-visible spectra (*λ_max_* = 664 nm) of the reaction mixture as a function of time (Figure 10). The intensity slowly decreased with time indicating slow degradation of the dye, and it was not completely degraded even after 4 h, as displayed by the large absorption maxima of the undegraded dye in Figure 10a. Now the photocatalytic degradation of an organic compound on the surface of a nanoparticle consists of two steps; in the first step, the compound is adsorbed on the surface of the nanoparticles, followed by a photocatalytic reaction in the second step [4]. The highly active surface of the T_sg_ nanoparticles was already occupied by adsorbed organic materials from the reaction mixture during synthesis, as revealed in the TGA experiment, which hindered the approach of MB to the nanoparticle surface. The adsorption study displayed in Figure 8 revealed that the sol-gel nanoparticles have zero or negligible adsorption ability for MB. Both factors might have contributed to the slow and incomplete degradation of the dye during that time.

The as-synthesized nanoparticles (T_sg_) were calcined for 2 h at 400 °C to clean the surface. In the presence of the calcined nanoparticles (T_sgc_), the intensity of UV-visible absorption maxima of the reaction mixture quickly declined revealing that the degradation was very fast at the early stage and it took about 3 h for complete degradation (the *λ_max_* became indistinguishable from the baseline) as displayed in Figure 10b. The increased crystallinity (108% increase) of the calcined particles might have contributed to the effectiveness of the degradation [38]. It is interesting to note that the photodegradation of MB catalyzed by the hydrothermal nanoparticles (T_ht_) was almost identical to that in the presence of calcined sol-gel nanoparticles (T_sgc_) as revealed in the UV-visible absorption profile of the reaction mixtures shown in Figure 10b and Figure 10c, respectively. This is because the surface of both the nanoparticles was almost clean (cf. Figure 3), and also, they both were an anatase polymorph of TiO_2_ having the same level of crystallinity (cf. Table 1). The degradation efficiency of the hydrothermal nanoparticles increased after calcination at 400 °C (T_htc_), as observed in the absorbance profile of the reaction mixture shown in Figure 10d, although its degree of crystallinity decreased upon calcination. This is because T_htc_ is a mix of the anatase and brookite phases of TiO_2_, which is well known to have much better photocatalytic efficiency than pure anatase or brookite due to a synergistic effect that facilitates charge separation and reduces electron-hole recombination [40,41,42]. This fact is also reflected in the large red shift of the absorption maxima of the nanoparticle dispersion in UV-visible spectra of T_htc_ in our study (cf. Figure 5).

#### 2.2.5. Photocatalytic Degradation of Anionic MO Dye under UV-B Irradiation

**Figure 11:**
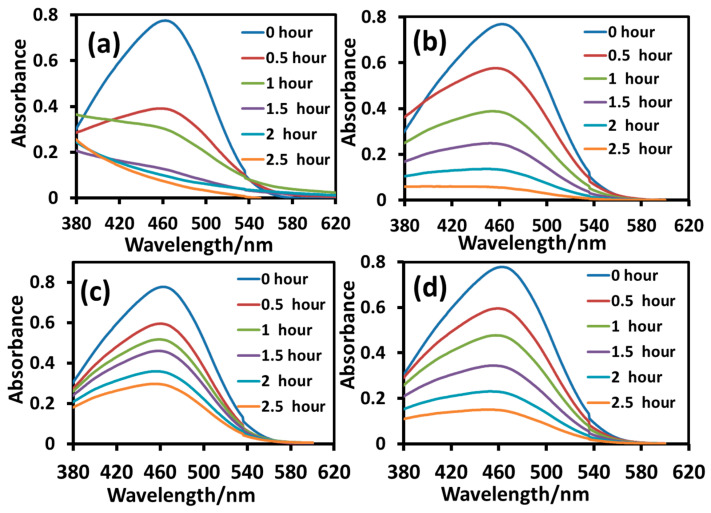
UV-visible absorption spectra of aqueous solutions of MO during degradation under UV-B irradiation in the presence of T_sg_ (**a**), T_sgc_ (**b**), T_ht_ (**c**), and T_htc_ (**d**). Reaction parameters: dye = 10 mg/L, nanoparticle = 8 mg/10 mL, temperature = 10 °C.

The photodegradation of anionic MO was conducted in the same set-up under identical conditions and the results are shown in Figure 11. The photocatalytic efficiency of the nanoparticles was determined from the intensity of the *λ_max_* (464 nm) of MO. The UV-visible absorption profiles of the reaction mixtures revealed that the photodegradation of MO was fast at the initial stage and it was almost complete within one and a half hours in the presence of the T_sg_ nanoparticles as displayed in Figure 11a, reflecting some attractive interaction of the dye with the nanoparticle surface due to the presence of the adsorbed organic materials on it. After calcination at 400 °C (T_sgc_), the nanoparticle surface became clean and the surface gained a negative character due to the lone pair electrons on the oxygen atoms of the TiO_2_ surface, creating a repulsive interaction with the negatively charged MO, as a result, the photocatalytic efficiency decreased [30] and it took almost three hours for complete degradation, as observed in Figure 11b. The adsorption study presented in Figure 9a and Figure 9b displaying a 16% and 0% adsorption of MO on T_sg_ and T_sgc_, respectively may be taken as a support of the photocatalytic behavior of the two nanoparticles. In the case of the as-prepared hydrothermal nanoparticles (T_ht_), the degradation efficiency was much slower relative to the as-prepared sol-gel (T_sg_) nanoparticles as revealed in Figure 11c. Even though the crystallinity decreased upon calcination, it made the hydrothermal nanoparticles (T_htc_) a somewhat better photocatalyst for degradation of MO, as the same level of degradation was achieved within two hours that took more than 3.5 h by T_ht_. This is because T_htc_ is an anatase-brookite mixed phase as revealed by XRD analysis, which is well known to have a much better photocatalytic efficiency than pure anatase or brookite [40,41,42], as explained in the previous section. However, near 100% degradation was not observed in the presence of the hydrothermal nanoparticles before or after calcination, which is in clear contrast with the efficiency of the as-prepared sol-gel nanoparticles T_sg_ that catalyzed the photodegradation of MB to near completion within one and a half hours.

#### 2.2.6. Degradation Study in the Absence of Light

**Figure 12:**
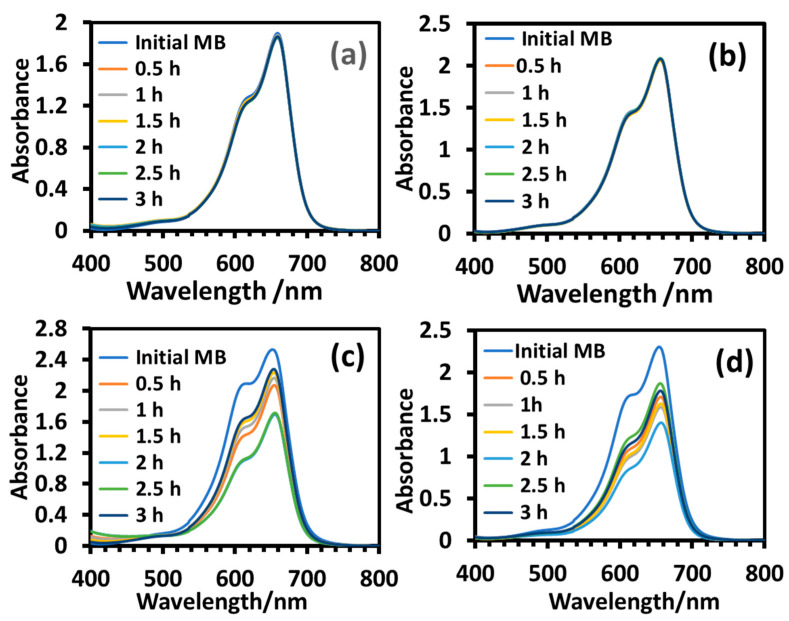
UV-visible absorption spectra of aqueous solution of MB during degradation under dark conditions in the presence of T_sg_ (**a**), T_sgc_ (**b**), T_ht_ (**c**), and T_htc_ (**d**). Reaction conditions: dye = 10 mg/L, nanoparticles = 8 mg/10 mL, temperature: 10 °C.

**Figure 13:**
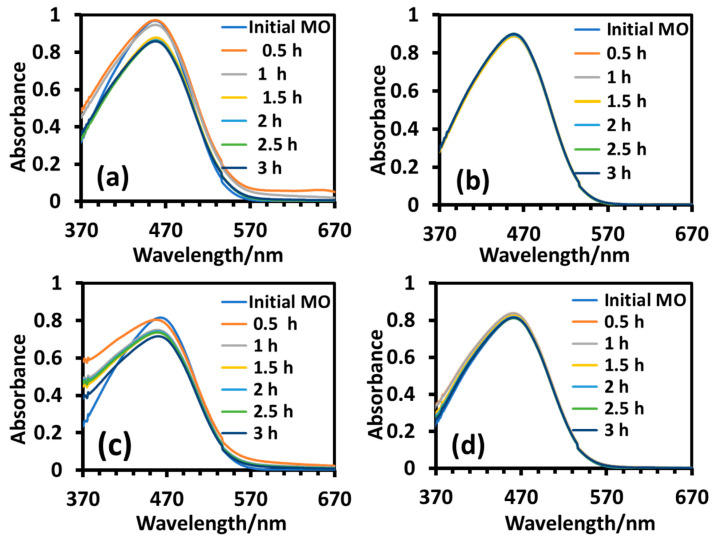
UV-visible absorption spectra of an aqueous solution of MO during degradation under dark conditions in the presence of T_sg_ (**a**), T_sgc_ (**b**), T_ht_ (**c**), and T_htc_ (**d**). Reaction parameters: dye = 10 mg/L, nanoparticle = 8 mg/10 mL, temperature = 10 °C.

The photocatalytic nature of the degradation of the model dyes by the synthesized nanoparticles was confirmed by carrying out the reaction between the dye and the nanoparticles under identical conditions but in the absence of any light (dark conditions) and the results are presented in Figure 12 for MB and in Figure 13 for MO. In the case of MB, the removal of the dye was 0% by the sol-gel nanoparticles T_sg_ and T_sgc_ whereas the maximum removal was 35% and 41% by the hydrothermal nanoparticles T_ht_ and T_htc_, respectively. The data are consistent with the adsorption study presented in Figure 8, revealing that the removal of the dye by the hydrothermal nanoparticles was solely due to adsorption. Also, the UV-visible spectra in Figure 12c,d were not consistent with time, presumably due to the uncertainty arising from the desorption of the dye from the nanoparticle surface during centrifugation and handling, revealing the physical nature of the adsorption. In the case of MO, a maximum removal of 10% was observed from corresponding UV-visible absorption spectra (Figure 13) for both the as-prepared sol-gel and hydrothermal nanoparticles T_sg_ and T_ht_, which was also inconsistent with reaction time; whereas the calcined nanoparticles T_sgc_ and T_htc_ did not show any removal efficiency. The results confirm that the removal of the dye by the nanoparticles under UV-B irradiation discussed in Section 2.2.4 and Section 2.2.5 was photocatalytic in nature.

#### 2.2.7. XRD Analysis of the Recovered Nanoparticles

**Figure 14:**
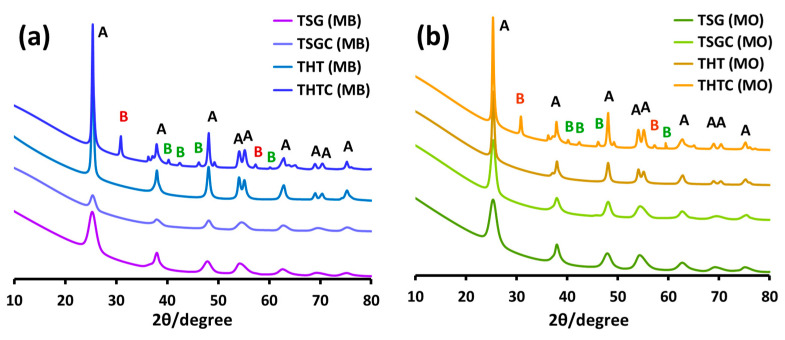
XRD patterns of the nanoparticles (indicated by the legends) recovered after the photocatalytic degradation reaction of MB (**a**) and MO (**b**) carried out under UV-B irradiation. Diffraction peaks corresponding to the anatase phase (A) and brookite phase (B) are also indicated. The brookite diffraction peaks indicated by the green color were absent in the fresh photocatalyst.

The nanoparticles were recovered after each photocatalysis experiment by centrifugation, repeatedly washed with ultrapure water and methanol, and dried in a vacuum oven at 50 °C. The stability of the photocatalysts was assessed by taking XRD of the recovered nanoparticles. The XRD patterns are displayed in Figure 14 and the crystallinity and crystallite size data obtained from XRD analysis are presented in Table 2.

It is revealed from Table 2 that the crystallite size of the nano-catalysts remained practically unchanged after the photocatalytic reactions. However, the effect of the harsh reaction condition on the crystallinity of the nanoparticles was mixed (cf. Table 1). In the case of the sol-gel nanoparticles, the trend in the change of crystallinity was the same irrespective of the dye photodegraded: crystallinity of the uncalcined nanoparticle dramatically increased while it decreased to an appreciable extent for the calcined species. In the case of hydrothermal nanoparticles, the crystallinity of the uncalcined species increased after the photodegradation reaction of MB while it slightly decreased after photodegradation MO. For the calcined hydrothermal nanoparticles, a huge increase in crystallinity was observed after the degradation of both the model dyes: 159% after MB degradation and 120% after MO degradation. From the results, it is inferred that the moderate energy UV-B radiation played a major role in altering the crystallinity of the photocatalyst species rather than the dye substrate it degraded. It is to be noted that the increase in the crystallinity in the photocatalyst was more pronounced for the species that had relatively lower levels of crystallinity at the outset. The huge (159%) increase in crystallinity obtained in T_htc_, a mix of the brookite and the anatase TiO_2_ nanomaterial, is reflected in the XRD pattern of the material by the appearance of several additional diffraction peaks characteristic of the brookite phase TiO_2_, which may indicate that the reaction condition favored the formation of the brookite phase of TiO_2_. Further study on the matter is in progress.

#### 2.2.8. Selectivity of the Prepared Nanoparticles towards Catalyzing the Photodegradation of MB and MO under UV-B Irradiation

**Figure 15:**
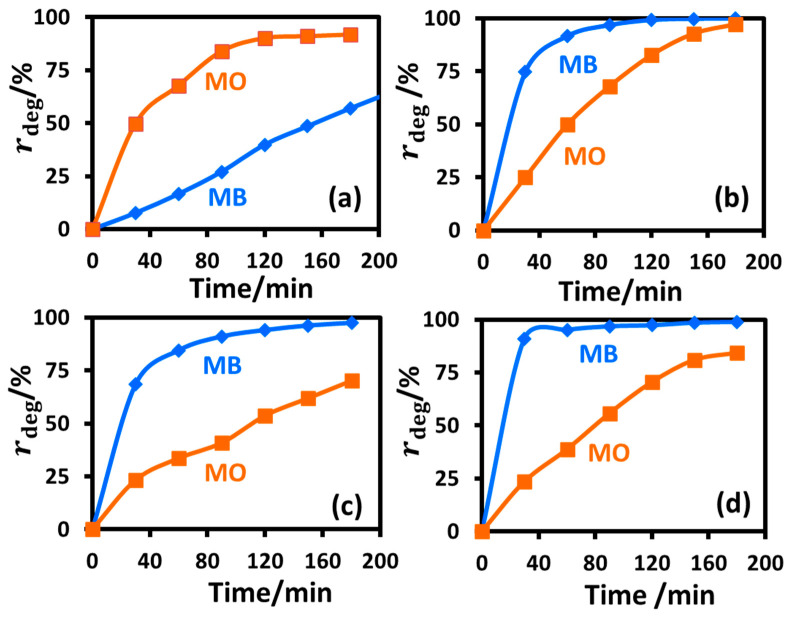
Comparison of the extent of degradation ($r_{deg}$) of MB and MO dyes in the presence of T_sg_ (**a**), T_sgc_ (**b**), T_ht_ (**c**), and T_htc_ (**d**) under UV-B irradiation. Reaction parameters: dye = 10 mg/L and nanoparticles = 8 mg/10 mL, temperature = 10 °C.

Figure 15 compares the results of the photocatalytic degradation efficiency of the TiO_2_ nanoparticles synthesized by the sol-gel and the hydrothermal synthetic routes, and also the effect of calcination of these nanoparticles on the degradation efficiency. The untreated TiO_2_ nanoparticles prepared by the sol-gel technique were remarkably selective in catalyzing the photodegradation of the model anionic dye MO over the cationic MB (90% for MO versus 40% for MB degradation in two hours) (Figure 15a). The said selectivity was reversed for the nanoparticles calcined at 400 °C, especially in the early stage of the degradation reaction (50% for MO versus 90% for MB in one hour) (Figure 15b). The as-prepared hydrothermal TiO_2_ nanoparticles displayed selectivity in the opposite sense of the as-prepared sol-gel nanoparticles (T_sg_) towards the degradation of the said model dyes: 41% degradation for MO versus 91% degradation for MB in one and a half hours (Figure 15c). Unlike the sol-gel particles, the selectivity towards degrading the model dyes did not alter for the hydrothermal nanoparticles after calcination (Figure 15d); however, the rate of conversion increased significantly due to improved crystallinity and formation of a mix of anatase and brookite phases in the photocatalyst upon calcination [38] that presumably decreased optical bandgap of the calcined materials, as discussed in Section 2.1 under Figure 5 and in Section 2.2.4 and Section 2.2.5 above.

For a better quantitative description of the photocatalytic specificity of the synthesized nanoparticles, the UV-visible spectroscopic data were utilized for analyzing the kinetics of the photocatalytic degradation of the dyes. The initial degradation reaction reasonably followed the apparent first-order kinetic path [30,43,44] according to Equation (2),

*ln*(*C*_0_/*C_t_*) = *kt* (2)

where *C*_0_ and *C_t_* are the initial concentration and the concentration of the substrate at time *t*, during the photocatalytic reaction under UV-B irradiation at 10 °C in the presence of the nanoparticles; *k* is the apparent first-order rate constant.

**Figure 16:**
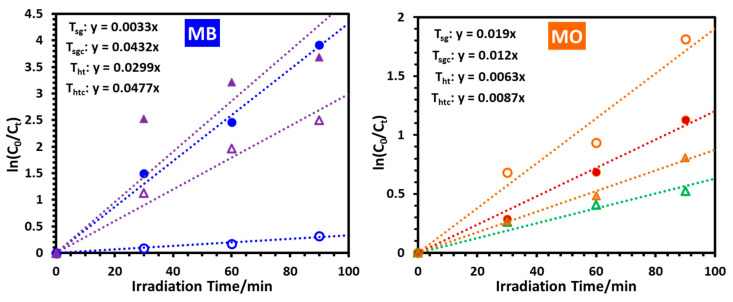
Apparent first-order kinetic plots prepared from UV-visible absorption data of the first ninety minutes of photocatalytic degradation of MB and MO under UV-B irradiation in the presence of nanoparticles T_sg_ (open circles), T_sgc_ (closed circles), T_ht_ (open triangles), and T_htc_ (closed triangles) at 10 °C.

The plots of *ln*(*C*_0_/*C_t_*) versus time (*t*) of UV-B irradiation are presented in Figure 16 and the values of the apparent first-order rate constant *k*, along with the half-life for the degradation of the model dyes are presented in Table 3. The selectivity of the untreated sol-gel TiO_2_ nanoparticle T_sg_ for catalyzing the photodegradation of MO over MB is also clear from the apparent t_1/2_ values of 36.5 min for MO versus 210 min for MB. For the calcined sol-gel nano-catalyst T_sgc,_ the t_1/2_ values are 57.8 min and 16.0 min for MO and MB, respectively, clearly revealing the reversal of the specificity. Both untreated (T_ht_) and calcined (T_htc_) hydrothermal nanoparticles were more selective for degrading the cationic dye MB than the anionic dye MO which is reflected in the t_1/2_ data, with T_htc_ having higher rate constants because it is a mix of the anatase and brookite phases [40,41,42].

## 3. Materials and Methods

### 3.1. Materials

Titanium tetraisopropoxide (TTIP) was obtained from Tokyo Chemical Industry Co. Ltd. (Tokyo, Japan). Analytical-grade chemicals such as aqueous ammonia solution (28 wt%), acetic acid, methanol (MeOH), ethanol (EtOH), MO, and MB were utilized without additional purification. Ultrapure water (UP) was obtained from a reverse osmosis water purification system, WPB-RO-15-UVF from MRC Laboratory Equipment Limited, Essex, UK.

### 3.2. Preparation of the TiO_2_ Nanoparticles

#### 3.2.1. Preparation of TiO_2_ Nanoparticles by Sol-Gel Technique

**Figure 17:**
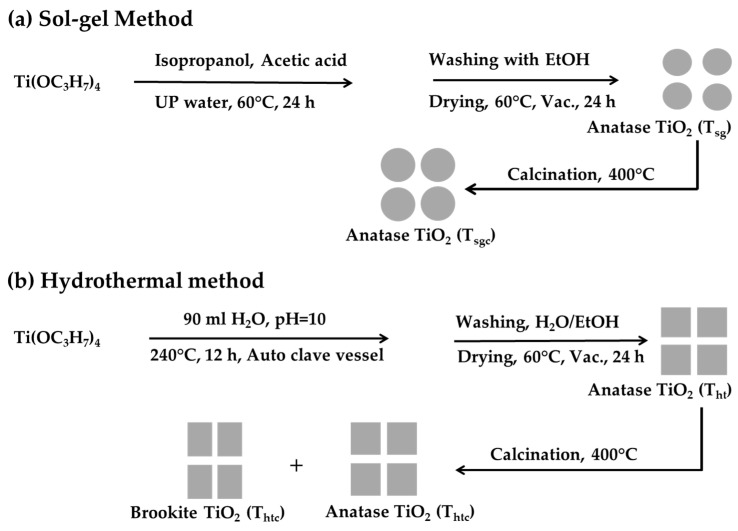
The schematic diagrams for the preparation of TiO_2_ nanoparticles from titanium tetraisopropoxide (TTIP) by: (**a**) sol-gel and (**b**) hydrothermal methods.

TiO_2_ nanoparticles were synthesized by the sol-gel method according to the scheme shown in Figure 17a. In a typical procedure, 9.68 g of TTIP were dissolved in 20 mL of isopropanol by stirring magnetically at 450 rpm for 20 min in a three-necked 500 mL round-bottom flask. Then acetic acid (1.2 mL) and ultrapure water (250 mL) were added into the TTIP solution, with the stirring continued. The reaction was carried out for 24 h at 60 °C. Following the reaction, the product was centrifuged and washed with EtOH. The centrifugation-redispersion washing procedure was repeated five times. Finally, the TiO_2_ nanoparticles were dried in a vacuum oven at 60 °C for 24 h. The product obtained was 1.978 g (73% yield). A portion of the product was calcined at 400 °C for 2 h in a muffle furnace.

#### 3.2.2. Preparation of TiO_2_ Nanoparticles by Hydrothermal Technique

TiO_2_ nanoparticles were also synthesized from TTIP following the hydrothermal technique, as described schematically in Figure 17b. Sodium hydroxide was used as the mineralizer and TTIP as the source material. In a typical procedure, 14.50 g TTIP was added to 50.0 mL of ultrapure water with vigorous magnetic stirring. A milky white sol was produced. The pH of the sol was adjusted to 10 by dropwise addition of an aqueous 1.0 M NaOH solution over several minutes. Finally, the sol was diluted to 90 mL by adding ultrapure water and was transferred to a 200 mL Teflon-lined autoclave jar. The jar was sealed in a stainless steel housing and was heated to 240 °C for 12 h in a thermostatic oven. Following the reaction, the product was purified from a 3:2 ultrapure water/EtOH medium by five consecutive centrifugation-redispersion cycles. Finally, TiO_2_ nanoparticles were dried at 60 °C for 24 h in a vacuum oven (3.1030 g, 76% yield). A portion of the product was calcined at 400 °C for 2 h in a muffle furnace.

### 3.3. Instruments

The surface Morphology of the nanoparticles was studied by a Scanning Electron Microscope (Model no. JSM-7610F from JEOL Ltd., Tokyo, Japan) at an accelerating voltage of 15 kV. Fourier-transform infrared (FTIR) spectra of the materials were taken on a PerkinElmer FTIR spectrophotometer, model number FTIR-100. Thermogravimetric analysis (TGA) of the materials was conducted on a PerkinElmer STA-6000 instrument at a heating rate of 10 °C/min under an N_2_ gas environment. The XRD of the samples was taken on a Rigaku, SmartLab-SE X-Ray Diffraction Analyzer. A Shimadzu (Kyoto, Japan) DR 1800 UV-visible spectrometer was used for optical absorbance measurement as a function of the wavelength in the range of 200–800 nm. A centrifuge machine (Model no. BKC-TH1611, Biobase Corporation, Jinan, China) operated at 10,000 rpm was used for the purification of the nanoparticles by repeated washing.

### 3.4. Adsorption and Degradation Experiments

An aqueous solution of MB or MO (10 mg/L) was mixed with either T_sg_ or T_sgc_ or T_ht_ or T_htc_ (8 mg/10 mL) and placed in an ultrasonic bath for 5 min and then kept in the dark for 30 min at ambient temperature (25 °C) under unstirred condition after which a portion of the mixture was centrifuged at 6000 rpm and UV-visible spectra of the supernatant was taken. The extent of adsorption was calculated from the difference in the UV-visible absorption of the supernatant from that of the initial dye solution.

For the degradation experiment, the sonicated dye/nanoparticle dispersion was taken in a double-walled Pyrex glass reaction vessel (100 mL) connected to a thermostatic water bath set at 10 °C. Before applying UV-B radiation, the reaction mixture was magnetically stirred in the dark for 30 min. Then, a UV light source (Quartz UV-B bulb, 3 w, 285 nm) was applied to the dye/nanoparticle dispersion for a desired period. The degradation reaction was followed by withdrawing 5.0 mL aliquots of the reaction mixture at regular intervals, centrifuging for 5 min at 6000 rpm, and measuring the absorbance of the supernatant with a UV-visible spectrophotometer. A degradation study of the model dye compounds was also carried out in an identical manner in the absence of light for comparison.

The extent of removal of the dye by adsorption or degradation in the presence of the TiO_2_-nanoparticles in a certain time $(r_{rem}$ in %) was calculated from the following Equation (3):

(3) $$r_{rem}=\frac{a_{0}-a_{t}}{a_{0}}\times 100\%$$

where $a_{0}$ and $a_{t}$ are the absorbances at times 0 and *t*, respectively.

## 4. Conclusions

TiO_2_ nanoparticles synthesized by sol-gel and hydrothermal routes and annealed (calcined) at 400 °C were characterized by standard methods. The catalytic efficiency of the nanoparticles for the photodegradation of two organic model compounds, cationic methylene blue (MB) and anionic methyl orange (MO), under UV-B (285 nm) irradiation was investigated. The untreated sol-gel nanoparticle (T_sg_) showed a higher selectivity for catalyzing photodegradation of MO than MB (90% versus 40% in two hours). This was attributed to an attractive interaction between anionic MO and the nanoparticle surface that adsorbed organic matter during synthesis acquiring a positive charge, facilitating photodegradation of the former. For the annealed nanoparticles (T_sgc_) the selectivity for photocatalysis reversed (50% for MO versus 90% for MB in one hour) as the surface of T_sgc_ was clean and negative due to the lone-pair electrons of oxygen of TiO_2_; it preferentially adsorbed and more efficiently degraded the cationic MB relative to MO. The surface of the hydrothermal nanoparticle (T_ht_) was clean at the outset because it was synthesized at an elevated temperature (240 °C) for a longer period, and also had a negative character. Consequently, it showed a photocatalytic selectivity for the cationic MB over MO, a behavior similar to the calcined sol-gel nanoparticle T_sgc_. The annealing of these particles (T_htc_) did not alter the nature of the surface and hence the photocatalytic selectivity for MB was sustained. However, T_htc_ being an anatase-brookite mixed phase showed better photocatalytic efficiency than the as-prepared T_ht_ for the photodegradation of both the model substrates.

## Figures and Tables

**Table 1:** The degree of crystallinity (*λ*) and crystal size (*l*) of the synthesized TiO_2_ nanoparticles.

Name of Samples	*λ*/%	*l* (± Error)/nm
T_sg_	17.8	4.39 ± 0.19
T_sgc_	37.1	6.19 ± 0.56
T_ht_	31.2	15.99 ± 0.91
T_htc_	17.0	19.88 ± 1.87

**Table 2:** The degree of crystallinity (*λ*) and crystal size (*l*) of the TiO_2_ nanoparticles recovered after the photocatalytic degradation reactions, calculated from the XRD data. The letters in parenthesis after the name of a nanoparticle represent the name of the dye it degraded by photocatalysis.

Name of Sample	*λ*/%	% Change in *λ*	*l* (±Error)/nm
T_sg_ (MB)	34.2	+92	4.97 ± 0.65
T_sgc_ (MB)	22.1	−40	5.98 ± 0.51
T_ht_ (MB)	40.9	+31	16.00 ± 0.91
T_htc_ (MB)	44.0	+159	23.48 ± 1.64
T_sg_ (MO)	34.2	+92	5.21 ± 0.56
T_sgc_ (MO)	31.5	−15	6.77 ± 0.52
T_ht_ (MO)	29.8	−4.5	15.83 ± 0.86
T_htc_ (MO)	37.4	+120	22.08 ± 1.56

**Table 3:** The values of the apparent first-order rate constant (*k*) and the half-life (t_1/2_) for the degradation of the model dyes MB and MO in the presence of nanoparticles T_sg_, T_sgc_, T_ht_, and T_htc_ under UV-B irradiation.

Sample Name	*k*_MB_ × 10^3^/min^−1^	*k*_MO_ × 10^3^/min^−1^	t_1/2_(MB)/min	t_1/2_(MO)/min
T_sg_	3.3	19.0	210	36.5
T_sgc_	43.2	12.0	16.0	57.8
T_ht_	29.9	6.3	23.2	110
T_htc_	47.7	8.7	14.5	79.7

## Data Availability

All data are included in the article.

## Sample Availability

Samples of the compounds are available from the authors.
